# The Cholera Pandemic, Still with Us after Half a Century: Time to Rethink

**DOI:** 10.1371/journal.pntd.0001003

**Published:** 2011-01-25

**Authors:** Edward T. Ryan

**Affiliations:** Massachusetts General Hospital and Harvard University, Boston, Massachusetts, United States of America

The recent outbreaks of cholera in Haiti, Pakistan, and Zimbabwe suggest that our current global action plans against cholera are failing. This issue contains two important articles that will help inform our discussions on ways to respond to the global cholera situation. Cholera is a severely dehydrating illness caused by *Vibrio cholerae*, a Gram-negative organism. *V. cholerae* exists in environmental aquatic reservoirs, and, as a result, cholera is not an eradicable disease, but it is controllable. Humanity has recognized seven cholera pandemics since 1817, all originating in Asia. The most recent pandemic began in 1961 in Indonesia, making it at half a century the longest cholera pandemic on record. As opposed to burning out after 5–20 years as all previous pandemics have done, this pandemic, if anything, seems to be picking up speed. Cholera outbreaks are occurring with increasing frequency and severity, as demonstrated by the recent major outbreaks in Nigeria, Angola, Pakistan, Vietnam, Zimbabwe, and now Haiti. This is on top of all the endemic infections that largely go “unnoticed”. In fact, cholera is now endemic in approximately 50 countries worldwide, and *V. cholerae* infects 3–5 million individuals each year, killing approximately 100,000, only a minority of whom die in outbreaks that garner media attention.

Cholera can kill a healthy person within 12–24 hours of onset of diarrhea and can cause explosive outbreaks; thus, it has the ignominious distinction of probably being the pathogen that can kill the most number of humans in the shortest period of time. Cholera outbreaks are associated with chaos, and they severely stress health care systems and communities. Humanity's response to cholera led to the development of oral rehydration solution (ORS) and evidence-based approaches to rehydration therapy. ORS perhaps represents the paradigmatic successful interface of basic science and biomedical science and a cost-effective, inexpensive public health intervention. ORS costs pennies, can be made locally or in a rural house, requires minimal or no training for production and administration, can be used in extremely adverse circumstances, and mitigates dehydrating illness and death for all causes of diarrhea, not just cholera. It is estimated that ORS has saved the lives of 40 million individuals since it was first endorsed by the World Health Organization (WHO) in the 1980s. In part because of this success of ORS, response efforts to cholera over the last 30 years have largely focused on treating individuals who become afflicted in the short-term, and trying to provide safe water and improved hygiene in the long-term. However, as we mark a half century for this pandemic, we must stop and ask: is this still the best approach?

Despite heroic efforts by many, 13% of the world's population still lacks access to safe water. To translate this statement, safe water would have to be provided every day for 10 years to an additional 240,000 people who currently lack safe water each day to eliminate this disparity. And this assumes that people with currently tenuous access to safe water do not slip backward, and that somehow we also provide safe water to the 1–3 billion people who will be joining us on the planet in the next few decades. As such, the provision of safe water to all of the world's population is truly a long-term solution, and one a realist would say will take decades. A second piece of data that needs to be considered as we mark the 50th anniversary of the start of this pandemic is that the causative agent of our current pandemic is different from those that caused the first six pandemics. *V. cholerae* O1 can be divided into two major biotypes. Earlier pandemics for which we have data were caused by what is referred to as the “classical” biotype, but the current pandemic is caused by the “El Tor” biotype. Compared to classical organisms, *V. cholerae* El Tor are much better at surviving in the environment, and are more likely to result in asymptomatic carriage in humans. The latter means that people can introduce the infection into a new zone unknowingly, and the former means that once a zone is involved, it may well become endemic for cholera. These facts may explain in part why our current pandemic extends much longer than all previous ones. It also means that outbreaks can be prolonged (as evidenced by Zimbabwe), and that there will be no quick fixes.

The El Tor variant has also undergone two major modifcations over the last 20 years. First, an El Tor O1 strain acquired a new lipopolysaccharide structure, forming a new variant serotype, O139. Since immunity to cholera is largely serotype specific, this meant that a new variant had evolved that could infect and kill individuals thought to be immune to cholera by previous exposure to O1. O139 spread rapidly in the 1990s through 11 Asian countries, but then was largely replaced by its cousin O1 El Tor again (for unclear reasons). More recently, El Tor has undergone another genetic event to create what is being referred to as a “hybrid” strain, an El Tor variant expressing classical cholera toxin. Cholera caused by the hybrid strain may be more clinically severe, and the hybrid is rapidly replacing the old El Tor strain in many areas. The prevalence of the hybrid strain may explain why we are seeing case fatality rates of 1%–5% (or higher) in recent outbreaks, as opposed to the <1% historically accepted as the goal for response teams.

With all this as background, are there other tools that we can bring to bear to control and respond to cholera? For instance, *V. cholerae* passaged through the human intestine is “hyperinfectious”, and this hyperinfectivity critically contributes to *V. cholerae*'s ability to cause explosive outbreaks. Could targeted or community-wide administration of antimicrobials in an initial phase of an outbreak sufficiently undermine this contributing factor to alter transmission dynamics? Such use of tetracycline in the 1970s was not beneficial, but would this still be the case if we used newer and more potent drugs requiring only single dose administration, such as azithromycin and doxycycline? What would be the trade-offs? Such an approach could easily drive drug resistance, but modeling analysis of the risk-benefit ratio of such an approach seems to at least be warranted. Any benefit would presumably only be temporary, and would not remove the need for a more comprehensive response. Few would argue that case detection, rehydration therapy, and provision of safe water and improved sanitation should be cornerstones of any integrated response, but should vaccination against cholera also be part of this response? Historically, the role of cholera vaccine has been controversial. Opponents to the use of cholera vaccine have largely said that in the chaos of a cholera outbreak, the majority of resources should focus on rehydration and provision of safe water and improved sanitation. The old parenteral cholera vaccine required multiple immunizations, had a high adverse event profile, and at best was moderately protective for few months. But with the development of improved (albeit not perfect) cholera vaccines, the emergence of prolonged outbreaks, and the endemic nature of El Tor cholera in so many areas of the world, it may be time to revisit this decision tree.

There are currently two oral cholera vaccines licensed and being manufactured in the world. Both are oral vaccines that contain killed *V. cholerae* organisms from different strains. One (Dukoral, Crucell) provides protection against *V. cholerae* O1 and contains a non-toxic B subunit of cholera toxin. It requires administration with buffer. The vaccine is approved for use over the age of 2 years, is WHO-pre-qualified, has been administered to over a million individuals, is safe and immunogenic, requires two or three administrations depending on age and previous exposure, and provides both direct and herd protection of 70%–90% in the 6 months following vaccination, and approximately 50% protection over 2–3 years. A bivalent O1 and O139 oral killed cholera vaccine is produced locally in Vietnam (mORC-VAX, VaBiotech,) and is currently being produced as Shanchol by Shantha Biotechnics in India for international distribution. Pre-qualification of the vaccine is being considered by the WHO. Shanchol and mORC-VAX are administered as 1.5 ml of fluid to be followed by safe water, is approved for use over the age of 1 year, is administered as one or two doses, and is as effective of Dukoral, with some suggesting that it may provide longer protection of up to 3–5 years, especially in endemic zones. It has not been field-evaluated in non-endemic zones.

The WHO position statements on the use of cholera vaccines have been evolving over the last 20 years. The initial position was that efforts should focus on treating patients with cholera and providing safe water and improved sanitation. The 2001 position statement suggested that cholera vaccine could be deployed as part of a program of response in endemic zones, but that oral killed cholera vaccine may have limited efficacy during epidemic or outbreak responses. The most recent WHO position statement of 2010 further suggests that cholera vaccine should be used in endemic settings or in predictable situations, and suggests that the vaccine could be considered in reactive situations (that is during an outbreak that has already started), but that data for such use are lacking and needed [1]. It is in this context that the two reports in the current issue of *PLoS Neglected Tropical Diseases* make significant contributions. The report by Reyburn et al. [2] describes the modeling of the effect of cholera vaccine once an outbreak has occurred using data from a number of recent outbreaks. The researchers modeled 50% and 75% vaccine coverage, with completion of vaccination ranging from “rapid” (10 weeks after an outbreak was first reported), to a “maximum” of completion of vaccination 33 weeks after an outbreak is first reported. The researchers found that even delayed responses could have benefit, and their model neither included herd effect modifiers, nor the effect that vaccination could have on subsequent disease burden after the initial outbreak has waned into an endemic situation. This issue also contains a report by Anh et al. [3] describing a case-control study of the reactive use of the Vietnamese killed cholera vaccine during a significant outbreak in Hanoi, an endemic zone. Administration of one or two doses of the vaccine was found to provide approximately 76% protective efficacy after controlling for additional factors. The only previous report of reactive cholera vaccine use was an observational study in Micronesia using an oral live attenuated cholera vaccine, and in that situation, the vaccine was also associated with approximately 80% protective efficacy.

These reports are significant, and will contribute to the discussion on the role that cholera vaccine could play in both short- and long-term response plans. What role cholera vaccine will play, if any, is still uncertain, and even if cholera vaccine is incorporated into response plans, many logistic hurdles would remain (who will pay, will vaccine be stockpiled, which vaccine would be used, who would control its use, delivery, and deployment, how will a vaccine program synergize with other response efforts and immunization efforts, etc.). But one thing is very clear as we mark the 50th anniversary of the start of our current war with cholera: we have a wily and adaptive foe that has changed the rules of engagement repetitively, and it may be time for us to similarly adapt our strategies.
